# New Insights into the Roles and Mechanisms of Spermidine in Aging and Age-Related Diseases

**DOI:** 10.14336/AD.2021.0603

**Published:** 2021-12-01

**Authors:** Yu-Qing Ni, You-Shuo Liu

**Affiliations:** ^1^Department of Geriatrics, The Second Xiangya Hospital, Central South University, Changsha, Hunan, China; ^2^Institute of Aging and Age-related Disease Research, Central South University, Changsha, Hunan, China

**Keywords:** spermidine, aging, age-related diseases, longevity, autophagy

## Abstract

High incidences of morbidity and mortality associated with age-related diseases among the elderly population are a socio-economic challenge. Aging is an irreversible and inevitable process that is a risk factor for pathological progression of diverse age-related diseases. Spermidine, a natural polyamine, plays a critical role in molecular and cellular interactions involved in various physiological and functional processes. Spermidine has been shown to modulate aging, suppress the occurrence and severity of age-related diseases, and prolong lifespan. However, the precise mechanisms through which spermidine exerts its anti-aging effects have not been established. In this review, we elucidate on the mechanisms and roles underlying the beneficial effects of spermidine in aging from a molecular and cellular perspective. Moreover, we provide new insights into the promising potential diagnostic and therapeutic applications of spermidine in aging and age-related diseases.

## 1. Introduction

Due to improvements in the quality of life, social security, and medical conditions, life expectancy has been significantly prolonged. However, as the global population ages, socioeconomic challenges are becoming increasingly common, which has attracted global attention. Aging is an inevitable and irreversible biological process that is characterized by a gradual and progressive loss of physiological integrity and functions. It is a predominant risk factor for higher incidences of chronic disorders such as cardiovascular diseases (CVDs), neurodegenerative diseases, metabolic diseases, musculoskeletal diseases and immune-senescence diseases [1, 2]. The elderly population often present with other morbidities that may eventually lead to death [3]. Studies on aging, including those focused on major age-related diseases, are still in their early stages [4]. Due to the rising aging population and the prevalence of age-related diseases, it is important to develop novel preventive and therapeutic interventions to suppress aging and decrease the burden of age-related diseases.

Polyamines are ubiquitous polycations that are found in all cells, tissues, and organs. They can interact with negatively charged molecules such as DNA, RNA, adenosine triphosphate, and proteins. These molecules exert multiple functions in many physiological and pathophysiological processes, including cell proliferation, differentiation, growth, tissue regeneration and gene regulation [5]. Due to its antioxidant functions, anti-inflammatory properties, enhanced proteostasis and improved mitochondrial metabolic functions, spermidine, a naturally occurring polyamine, is involved in a series of biological events, including autophagy induction, apoptosis, transcription, and DNA stability [4, 6]. The concentration of spermidine declines with age, and exogenous spermidine supplementation reverses age-associated adverse changes and prolongs the lifespan [7]. Spermidine is associated with longevity [8, 9]. Given that it interacts with various molecules, spermidine influences aging through diverse mechanisms. However, the roles and mechanisms through which spermidine modulates the process of aging and alters the course of age-related diseases have not been elucidated. Therefore, in this review, we compile and update the latest knowledge regarding how spermidine modulates aging and reveal its potential diagnostic and therapeutic applications in age-related diseases.

**Figure 1.**
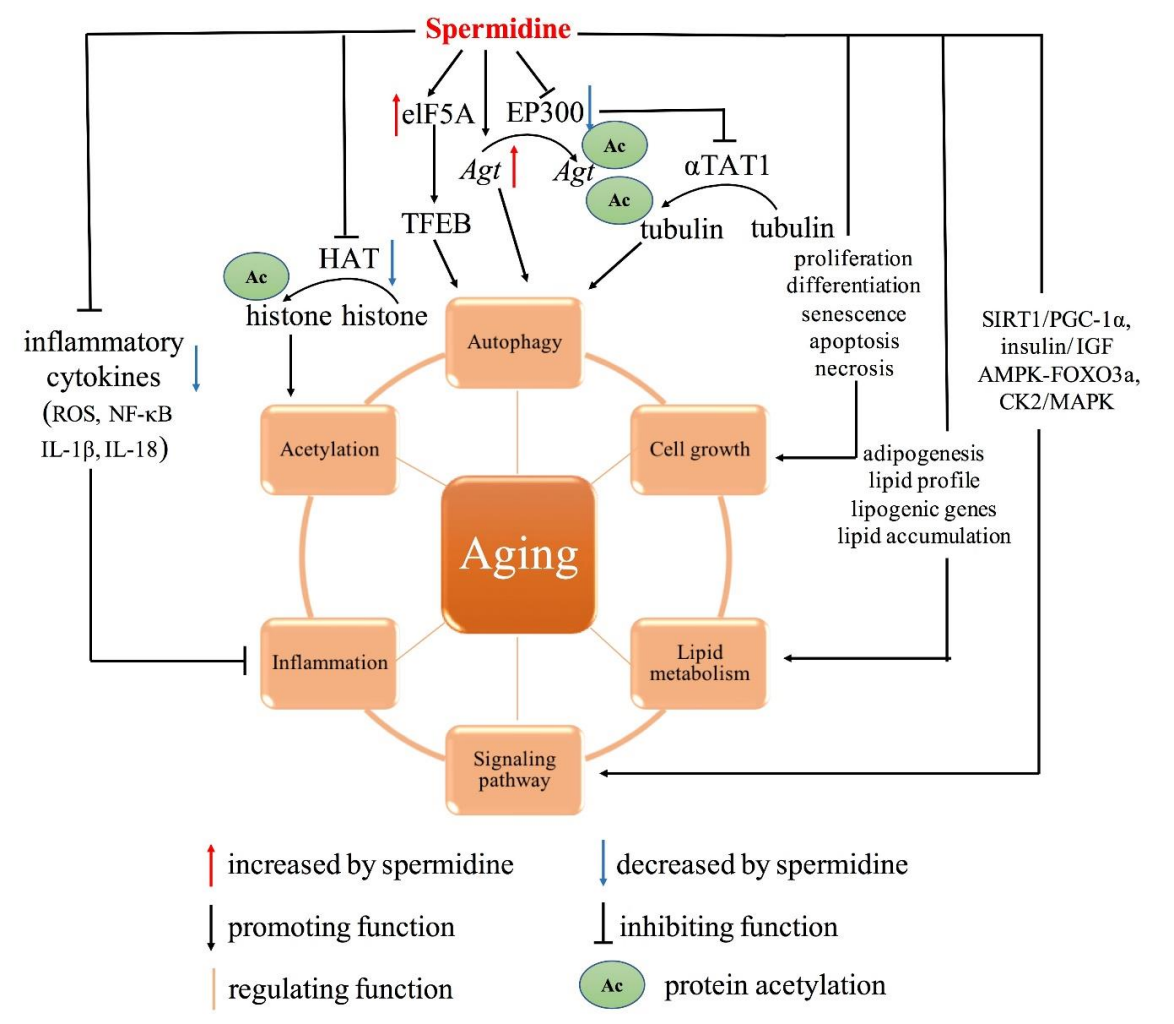
**Molecular and cellular mechanisms of spermidine in age-related diseases.** Spermidine is an inducer of autophagy, which is the main mechanism of anti-aging. First, spermidine triggers autophagy by modulating the expressions of *Atg* genes. Second, it regulates transcription factor elF5A to promote the synthesis of transcription factor TFEB. Third, spermidine inhibits EP300, which directly promotes the acetylation of Atg genes and indirectly stimulates deacetylation of tubulin due to inhibition of aTAT1. Besides, spermidine exerts potent anti-inflammatory roles by suppressing of multiple inflammatory cytokines, such as ROS, NF-κB, IL-1β and IL-18. Moreover, it is involved in regulation of cell proliferation, differentiation, senescence, apoptosis and necrosis, ultimately promoting cell growth and inhibiting cell death. As an anti-aging agent, spermidine suppresses histone acetylation. Moreover, spermidine regulates lipid metabolism. On the one hand, it promotes the differentiation of preadipocytes into mature adipocytes. On the other hand, it alters lipid profile, modulates lipogenic gene expressions, and represses lipid accumulation. Furthermore, spermidine can delay aging through specific signaling pathways, such as SIRT1/PGC-1α, insulin/ IGF, AMPK-FOXO3a, and CK2/MAPK signaling pathways. Abbreviations: *Atg*: autophagy-related genes; aTAT1: a-tubulin acetyltransferase 1; EP300: E1A-associated protein p300; ROS: reactive oxygen species; NF-κB: nuclear factor kappa-B; IL: interleukin; SIRT1: Sirtuin-1; PGC-1α: peroxisome proliferator-activated receptor gamma coactivator alpha; IGF: insulin-like growth factor; AMPK: AMP-activated protein kinase; MAPK: mitogen-activated protein kinase

## 2. Mechanisms of spermidine in aging

Even though aging is inevitable, it can be modified by biological and genetic interventions, pharmaceuticals, lifestyle, and the systemic environment [10-12]. Spermidine has been shown to be important for prolonging survival outcomes, and abnormal changes in spermidine levels are associated with aging as well as disease development [13]. Intracellular concentrations of spermidine are suppressed during aging. Exogenous spermidine supplementation has been shown to extend the lifespans of flies, nematodes and yeast [14]. Moreover, a diet enriched in spermidine was shown to prolong the lifespans of mice [15]. However, studies on the mechanisms of action of spermidine are rare. Autophagy is the main mechanism of spermidine in delaying aging and prolonging the lifespan. In addition, spermidine exerts its effects through other mechanisms, including anti-inflammation, histone acetylation reduction, lipid metabolism and regulation of cell growth and signaling pathways [16]. In this section, we update on the field of spermidine research and discuss the potential mechanisms of spermidine in aging (Fig. 1).

### 2.1 Autophagy

Autophagy, an intracellular degradation system, is a complex process that delivers damaged or unnecessary cytoplasmic components into lysosomes [17, 18]. It can be divided into microautophagy, macroautophagy, and chaperone-mediated autophagy. The term autophagy is often used in reference to macroautophagy, the most common process through which cytoplasmic contents can be sequestered within the autophagosome, subsequently fusing with a lysosome or vacuole [19]. At basal levels, it is a process that is necessary to mediate proper cellular function, but can be adjusted by certain stimuli, such as aging, oxidative stress, or inflammation [20]. Aging enhances the formation of damaged cellular constituents, including proteins and organelles [21, 22], and suppresses cellular ability to degrade these components [23, 24]. Therefore, autophagy plays an important role in anti-aging and in improving longevity. Autophagy is primarily a cytoprotective mechanism [25, 26]. Induction of autophagy prolongs the lifespan, while its deficiency shortens the lifespan [27].

Spermidine has been shown to induce autophagy in multiple organs, including liver, heart, and muscle in mice [28], as well as in aging yeast, worms, flies, and cultured mammalian cells [14, 29]. Spermidine induces autophagy by adjusting the expression levels of autophagy-related genes (*Atg*). The *Atg* genes, such as *Atg* 7, *Atg* 15, and *Atg* 11 were up-regulated upon spermidine supplementation [8], while *Atg* gene knockout abolished spermidine-induced lifespan extension [14, 30]. Second, spermidine regulates autophagy by inducing the expression of transcription factor, elF5A, to increase the synthesis of transcription factor, TFEB [31]. Third, spermidine initiates autophagy by inhibiting protein acetylation [32]. The E1A-associated protein p300 (EP300) is an acetyltransferase that directly promotes acetylation of multiple autophagy-essential proteins, and indirectly stimulates tubulin deacetylation by inhibiting a-tubulin acetyltransferase 1 (aTAT1) [33, 34]. Spermidine enhances the deacetylation by reducing the expression of EP300. Besides, spermidine decreases acetylation by reducing the availability of acetyl-CoA [29]. Moreover, spermidine can also induce autophagy through other pathways, including regulation of inflammation and lipid metabolism among others [35, 36]. Altogether, autophagy is the most important mechanism through which spermidine exerts its anti-aging effects.

### 2.2 Anti-inflammation

Inflammation is a double-edged sword. It plays a crucial role in immunity by resisting pathogenic invasion. However, it may disrupt the balance of organisms, which may eventually lead to disease. Excessive inflammatory responses, also referred to as “inflammatory aging”, is a major risk factor for aging [37-39]. In addition, elevated expression levels of pro-inflammatory biomarkers, C-reactive protein (CRP), tumor necrosis factor-α (TNF-α), and interleukin-6 (IL-6) have been associated with a risk of developing various age-related diseases including cardiovascular diseases, cerebrovascular diseases, chronic kidney diseases, and metabolic syndrome [40-42].

Systemic effects of spermidine play a crucial role in delaying aging, possibly through its involvement in anti-inflammatory processes. In this section, we elucidate on the potential mechanisms through which spermidine suppresses inflammation. Eisenberg *et al*. reported that spermidine supplementation reduces chronic inflammation by decreasing TNF-α expression levels, thereby suppressing the occurrence and progression of cardiovascular dysfunctions [15]. Besides, Jeong *et al*. proved that spermidine exerts potent anti-inflammatory effects through various mechanisms [43]. First, it inhibits the accumulation of reactive oxygen species (ROS) and translocation of nuclear factor-kappa B (NF-κB). Second, spermidine is associated with the inhibition of inflammation related migration of immune cells. Spermidine supplementation has been shown to suppress protein expression levels of IL-1β and IL-18 [44]. These findings indicate that anti-inflammation is essential for spermidine-mediated delay of aging.

### 2.3 Cellular lifecycle

Cellular lifecycle involves proliferation, differentiation, senescence and apoptosis, which are structural and functional bases for organism growth, development, aging and death, respectively. Most cells exhibit a normal lifecycle, while a few cells deviate from a normal lifecycle due to interference of certain factors, including damage, necrosis or cancer. Dysregulated cellular lifecycle has been implicated in the pathogenesis of aging and age-related diseases. Spermidine is involved in regulation the lifecycle of cells [45]. With the relevant cumulative findings, herein we discuss the correlation between spermidine and cellular lifecycle.

#### 2.3.1 Spermidine and cell proliferation

Cell proliferation, which is attributed to cell division, is an important characteristic of living organisms. Cell cycle is responsible for cell growth, survival and death [46]. Spermidine plays a causative role in modulating the cell cycle [47], with small amounts of spermidine shown to sustain normal cell cycles [48]. Landau *et al.* reported that the absence of spermidine can cause growth cessation at the G_1_ phase by affecting the expression of cell cycle regulators [49]. Spermidine was also shown to enhance the proportion of S phase cells and maintain mitochondrial membrane potential, thereby improving the senescence of mouse neuroblastoma cells [50].

#### 2.3.2 Spermidine and cell differentiation

Cell differentiation refers to process through which cells from the same source gradually produce cell groups with different morphological structures and functional characteristics. Recent studies have revealed that spermidine is involved in cell differentiation [51, 52]. Emerging evidence indicates a role for spermidine in enhancing differentiation in differentiated chondrocytes and in adult stem cells [53]. Cervelli *et al.* proved that exogenous supplementation of spermidine impacts on D-gal-induced aging-related skeletal muscle atrophy during skeletal muscle differentiation [54].

#### 2.3.3 Spermidine and cell senescence

Cell senescence is characterized by cessation of replication, loss of proliferation potential, resistance to apoptosis, and increased protein production [55, 56]. Spermidine prevents cell senescence [57]. Elevated spermidine levels were associated with improved functions of “old” B cells, which might reverse immune aging [58]. Zhu *et al*. demonstrated that spermidine inhibits high glucose and neurotoxicity-induced senescence induced by upregulating the expression of cannabinoid receptor type 1 [59]. Suppressed p21 and p16 expression levels and senescence-associated β-gal staining indicated that spermidine improved bleomycin-stimulated premature cell senescence [60].

#### 2.3.4 Spermidine and cell death

Physiological cell apoptosis and pathological cell necrosis are collectively referred to as cell death. Cell apoptosis is a basic biological phenomenon of cells, which plays an important role in the removal of unwanted or abnormal cells from multicellular organisms. It is involved in the evolution of organisms, maintaining the stability of the internal environment and in the development of multiple systems. Spermidine can modulate cell apoptosis. The interaction between spermidine and mitochondrial membrane induces the release of cytochrome C, which is the prolusion to apoptosis [61]. In addition to interfering with the cell cycle, spermidine has also been found to slow down the aging process by preventing apoptosis [49, 50]. Cell necrosis refers to cell death under the induction of extreme physical, chemical or other serious pathological factors. The role of spermidine in cell necrosis has been reported [62]. Elevated spermidine levels were shown to suppress cell necrosis, prolong the lifespan and improve health in aging yeast [14].

### 2.4 Histone acetylation

Post-translational modification of histone has been shown to play a significant role in epigenetic changes during aging, with histone acetylation being the most important. Spermidine inhibits histone acetylation, thereby, exerting anti-aging effects [63, 64]. However, the mechanisms through which spermidine affects histone acetylation have not been fully elucidated. Alterations in spermidine concentrations impact on the activities of histone acetylases and deacetylases. Spermidine leads to hypoacetylation by decreasing histone acetylases rather than increasing histone deacetylases [8]. Eisenberg *et al.* reported that spermidine administration induces histone H3 deacetylation by inhibiting acetyltransferases (HAT) in yeast [14]. Burgio *et al.* documented that spermidine modulates histone acetylation levels by activating P/CAF, a highly conserved HAT, in vivo and in vitro [65]. These findings show that spermidine plays a crucial role in anti-aging by inhibiting histone acetylation.

### 2.5 Lipid metabolism

Lipid metabolism is a significant biochemical process that is involved in the synthesis and degradation of lipids, such as steroids, triglycerides, and phospholipids, to produce energy and maintain normal biological functions [66]. Aging is closely associated with lipid metabolism. Disruption of lipid metabolism has detrimental outcomes on health and longevity. A correlation between spermidine and lipid metabolism in aging has been reported [67].

Minois *et al.* found that increased triglycerides levels as well as altered phospholipid profiles and fatty acids were associated with extended lifespans in spermidine-fed flies [68]. Further research revealed that most of these spermidine-induced changes are largely regulated through autophagy. Gao *et al.* proved that spermidine regulates lipid metabolism through suppressing the expression of lipogenic genes via an AMP-activated protein kinase (AMPK) signaling pathway [69]. Moreover, spermidine has been shown to suppress necrotic core formation and lipid accumulation by stimulating cholesterol outflow [36]. Ma *et al.* noted that spermidine feeding reduces plasma lipid profiles and fat mass without affecting body weight, thereby exerting an potential effect in treating obesity [70]. Spermidine/spermine N1-acetyltransferase (SAT1) acetylates spermidine and spermine to generate N1-acetylspermine, N1,12-diacetylspermine, and N1-acetylspermidine. SAT1 activation is closely associated with beige adipocyte biogenesis and low-grade inflammation [71].

In addition to the complex interactions between spermidine and lipid metabolism, spermidine promotes the differentiation of pre-adipocytes into mature adipocytes at the cellular level. α-difluoromethylornithine (DFMO) is a catalytic suicide inhibitor of polyamine synthesis that decreases the expression of transcription factors critical to late adipocyte markers and pre-adipocyte differentiation [72]. Exogenous supplementation of natural spermidine was shown to initiate the differentiation of preadipocytes into mature adipocytes in the presence of DFMO, thereby regulating adipogenesis [73]. Collectively, the role of spermidine in lipid metabolism is one of the important mechanisms of its anti-aging effects.

### 2.6 Signaling pathways

Multiple signaling pathways are involved in modulation of aging and age-related diseases. Spermidine interacts with various signaling pathways to regulate the aging process [8]. However, the specific mechanisms have not been established. In this section, we elucidate on the relationship between spermidine and specific signaling pathways in aging.

Sirtuin-1/peroxisome proliferator-activated receptor gamma coactivator alpha (SIRT1/PGC-1α) signaling pathway is a major modulator of mitochondrial function and a vital contributor to aging and cardiovascular diseases. Wang *et al.* confirmed that spermidine stimulates mitochondrial biogenesis through the SIRT1/PGC-1α pathway and could, therefore, be used to prevent cardiac function degradation during aging [74]. In drosophila, dietary spermidine supplementation was associated with extended lifespan by suppressing insulin/ insulin-like growth factor (IGF) signaling [75]. FOXO3a, a downstream effector of AMP-activated protein kinase (AMPK), which is involved in the aging process, has been associated with longevity [76, 77]. Fan *et al.* showed that spermidine protects against aging-related skeletal muscle atrophy by suppressing apoptosis and enhancing autophagy through the mediation of the AMPK-FOXO3a signaling pathway [78]. The ubiquitous kinase, CK2, has been reported to translate information in the mitogen-activated protein kinase (MAPK) pathway by detecting spermidine levels [79]. Moreover, spermidine upregulates the expression of MAPK family genes and to regulate MAPK phosphorylation [79, 80]. In conclusion, spermidine exerts its anti-aging properties by activating or suppressing signaling pathways.

### 2.7 Others

In addition to the above mechanisms through which spermidine modulates aging, biological functions of spermidine in protecting replicating DNA from oxidative damage have also been proposed. Oxidative damage by singlet oxygen, 1O_2_, leads to harmful effects on cells. Spermidine, as a positively charged molecule, can bind and precipitate DNA [81]. Khan *et al*. documented that spermidine protects DNA against oxidative attack, ensuring the integrity of DNA and RNA, thereby guaranteeing protein synthesis [82].

## 3. The role of spermidine in age-related diseases

**Figure 2.**
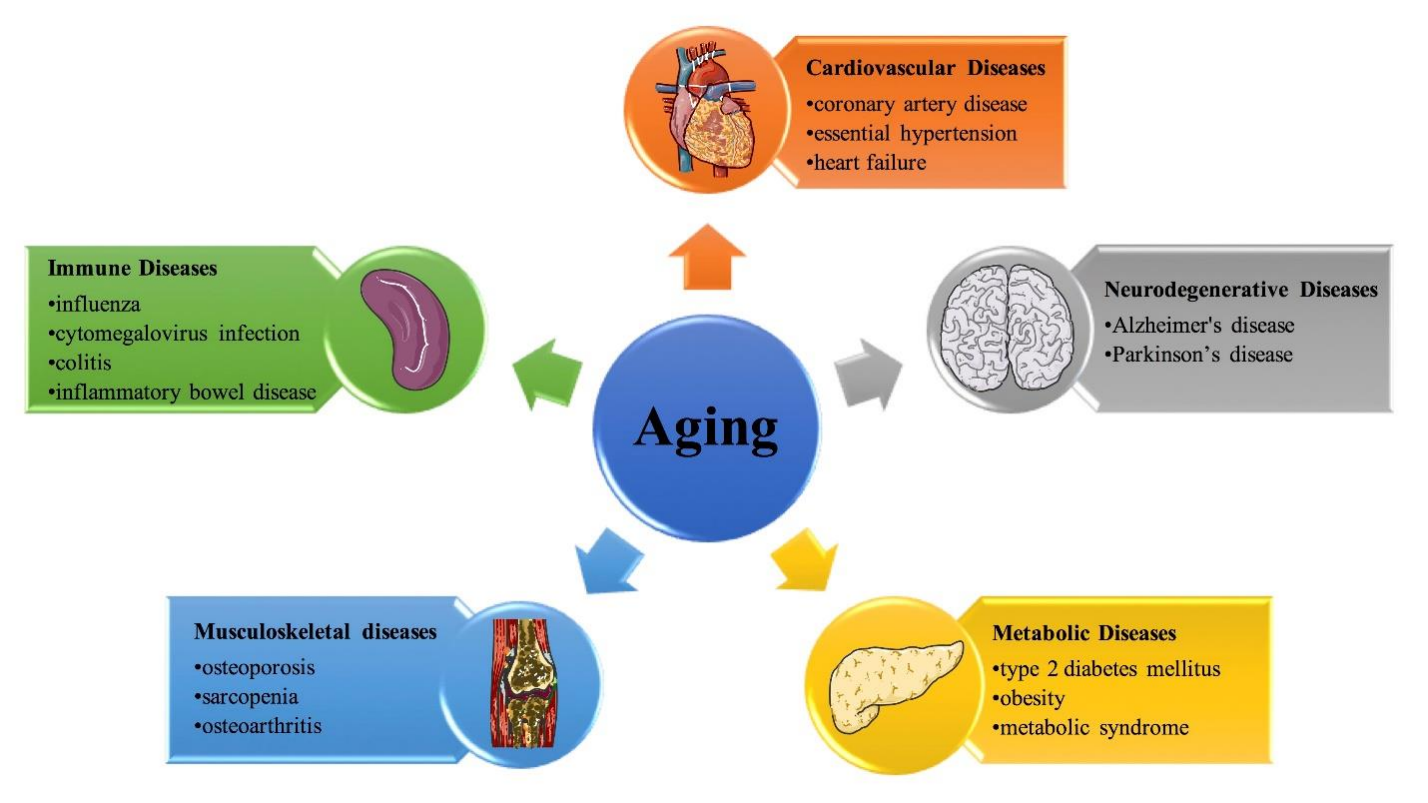
**The role of aging in age-related diseases.** This figure shows examples of age-related diseases where aging is one of the main risk factors.

Aging refers to a gradual deterioration of functionality and physiological integrity processes, which enhances susceptibility to age-related diseases (Fig. 2). Since the aging population is rapidly increasing, there is a need to thoroughly understand aging and age-related diseases. Sudies have reported on the mechanisms involved in aging, especially vascular aging [1, 2, 83-90]. Spermidine is a critical factor in aging and age-related diseases, including CVDs, neurodegenerative diseases, metabolic diseases, musculoskeletal diseases, and immune diseases. In this section, we discuss on the role of spermidine in age-related diseases (Table 1).

### 3.1 Spermidine and age-related CVDs

Aging is a major risk factor for the development of CVDs, which is a major cause of disability and death in the elderly population. Spermidine prevents against cardiac aging by improving left ventricular elasticity, diastolic function, and mitochondrial function [15]. It has been reported that CVDs such as coronary artery disease (CAD), essential hypertension (EH), and heart failure (HF) are highly influenced by spermidine levels [91].

#### 3.1.1 Spermidine and CAD.

CAD, one of the primary CVDs, is caused by the narrowing or blocking of vascular lumen due to atherosclerotic lesions in coronary arteries. Atherosclerosis (AS) is the major cause of CAD, which is a leading cause of mortality, especially in the elderly population. Studies have reported on the role of spermidine in CAD. Han *et al.* found an association between spermidine and myocardial ischemic reperfusion [92]. A prospective study found an inverse parallel relationship between spermidine and AS [93]. Tyrrell *et al.* documented that spermidine supplementation in aged mice inhibits AS via decreasing inflammatory cytokines and improving mitochondrial functions [94]. The risk of AS can also be reduced by the role of spermidine in autophagy [95, 96]. Besides, spermidine attenuates AS due to its antagonistic action on platelet aggregation, which is regarded as a causative factor for AS [96]. Plasma hyaluronan-binding protein (PHBP) is a factor VII activating protease involved in the modulation of vascular function, inflammation, and AS. Spermidine promotes the conversion of PHBP from a single-chain to a two chain form, thereby protecting against AS development [97]. In type 2 diabetes mellitus (T2DM), restoration of endothelial nitric oxide synthase (eNOS) activation by spermidine was found to be blocked by autophagy inhibitors, resulting in AS [98].

#### 3.1.2 Spermidine and EH

EH is characterized by increased vascular resistance, due to endothelial dysfunction and vascular remodeling, representing age-related functional and structural alterations, respectively [99]. Spermidine attenuates the development of EH during aging. Maione *et al.* reported on the beneficial effects of spermidine on N-methyl-D-aspartate (NMDA) induced EH [100]. Eisenberg *et al*. proved that dietary spermidine reduces high blood pressure by improving age-related diastolic [101]. Ornithine decarboxylase (ODC) is a crucial enzyme in the polyamine biosynthesis. In hypertensive tissues, spermidine concentrations have been shown to increase in tandem with alterations in ODC activity [102]. Ibrahim *et al*. proved that spermidine regulates blood pressure because it is an essential component of the blood pressure effect of angiotensin II [103].

#### 3.1.3 Spermidine and HF

HF is a clinical syndrome of aging-related phenotypes. An association between spermidine and HF has been reported. Appropriate induction of autophagy by spermidine might be involved in resistance to HF [104]. Moreover, spermidine supplementation was shown to prevent cardiac hypertrophy and protect cardiomyocytes, thereby delaying HF progression [15, 101]. Mitochondria are crucial in myocardial maintenance and development. Spermidine attenuates mitochondrial dysfunction during aging, which is the primary cause of HF development [105]. Wirth *et al.* found that the cardioprotective effect of spermidine at the histological level was associated with reduced telomere attrition in cardiac tissues [106]. Moreover, Tantini *et al.* indicated the effect of spermidine on the apoptosis of myocardial ischemic cells, which inhibited HF development [107].

**Table 1:** Roles of spermidine in aged-related diseases.

	Disease	Functions	Potential mechanisms
Cardiovascular Diseases	CAD	regulate myocardial ischemic reperfusion	modulate arterial blood perfusion [92]
	CAD	inhibit AS	decrease inflammatory cytokines and improving mitochondrial function [94]
	CAD	reduce AS	induce autophagy [95, 96]
	CAD	attenuate AS	antagonize platelet aggregation [96]
	CAD	protect from AS	promote conversion of PHBP [97]
	CAD	inhibit AS	activate eNOS [98]
	EH	inhibit EH	regulate NMDA and its receptors [100]
	EH	reduce blood pressure	improve age-related diastolic [101]
	EH	inhibit EH	regulate angiotensin II [103]
	HF	delay HF	prevent cardiac hypertrophy and protect cardiomyocytes [15, 101]
	HF	attenuate HF	attenuate mitochondrial dysfunction [105]
	HF	protect cardiac	reduce telomere attrition [106]
	HF	protect cardiac	regulate apoptosis of myocardial ischemic cells [107]
Neurodegenerative Diseases	AD	reduce memory decline	induce autophagy [110, 111]
	AD	ameliorate dementia	prevent inflammation and apoptosis of nerve cells [44]
	AD	influence memory	stimulate neural actions [112]
	PD	protect from PD	maintain the mitochondria in dopaminergic neurons function [113]
	PD	protect against PD	induce autophagy [114]
	PD	protect against PD	trigger PINK1-PDR1-dependent mitophagy [115]
	PD	alleviate PD	inhibit α-synuclein and promote climbing activity [116, 117]
	PD	protect against PD	exert anti-inflammatory and antioxidant properties [118]
	PD	attenuate PD	regulate SAT1 activity [119]
Metabolic Diseases	T2DM	prevent T2DM	improve insulin sensitivity and maintain glucose homeostasis [120]
	T2DM	prevent T2DM	promote facultative cell proliferation and maintain glucose homeostasis [121]
	T2DM	prevent diabetic complications	inhibit lipid peroxidation, hemoglobin glycation [122]
	T2DM	reduce hyperglycemic	enhance glucose utilization [123]
	T2DM	reduce nephropathy complications	reduce renal collagen [125]
	Obesity	ameliorate obesity	reduce adiposity and hepatic fat accumulation [120]
	Obesity	loss of weight	regulate lipid metabolism, inflammatory response, and thermogenesis [70]
	Obesity	attenuate obesity	induce autophagy in white adipose tissue [126]
	Obesity	alleviate obesity	enhance intestinal barrier function and alternate microbiota composition [127]
	Obesity	reduce adiposity	inhibit lipogenic genes expression [69]
	Obesity	attenuate obesity	increase energy expenditure [128]
	Metabolic syndrome	correct metabolic syndrome	activate TETA [129]
	Metabolic syndrome	inhibit metabolic syndrome	ameliorate hepatic steatosis and adipose tissue inflammation [70]
Musculoskeletal Diseases	Osteoporosis	enhance bone strength	promote warmth regeneration [130]
	Osteoporosis	prevent bone loss	disturb osteoclastic activation [131, 132]
	Osteoporosis	reduce migration and osteoclastogensis	inhibit RANKLE-mediated signaling pathway, prevent transcription factors [133]
	Sarcopenia	ameliorate skeletal muscle atrophy	regulate skeletal muscle differentiation [54]
	Sarcopenia	ameliorate muscle defects	induce autophagy [135]
	Osteoarthritis	improve osteoarthritis	activate autophagy [136]
	Osteoarthritis	alleviate synovitis, osteophyte formation and cartilage degeneration	inhibit TNF-α induced NF-κB/p65 signaling pathway [137]
	Osteoarthritis	protect chondrocytes	reduce oxidant and inflammatory responses [138]
Immune Diseases	influenza	improve CD8+ T cell responses	induce autophagy [140]
	cytomegalovirus infection	improve CD8+ T cell responses	induce autophagy [140]
	colitis	attenuate pathology	promote homeostasis differentiation of regulatory T cells [138]
	IBD	attenuate inflammation	induce autophagy [141]

CAD: coronary artery disease; AS: atherosclerosis; EH: essential hypertension; HF: heart failure; AD: Alzheimer's disease; PD: Parkinson’s disease; T2DM: type 2 diabetes mellitus; PHBP: plasma hyaluronan-binding protein; eNOS: endothelial nitric oxide synthase; NMDA: N-methyl-D-aspartate; SAT1: Spermidine/spermine N1-acetyltransferase; TETA: Triethylenetetramine dihydrochloride; TNF-α: tumor necrosis factor-α; NF-κB: nuclear factor kappa-B

### 3.2 Spermidine and age-related neurodegenerative diseases

Neurodegenerative diseases are featured by a progressive loss of selective populations of vulnerable neurons, and they can be classified as Alzheimer's disease (AD), Parkinson’s disease (PD), or motor neuron disease according to clinical characteristics. Spermidine protects against neuronal cell damage by inducing autophagy [108]. Therefore, supplementation with spermidine inhibits multiple neurological pathologies including neurodegeneration, memory loss, cognitive decline, and motor impairment in aging [109].

#### 3.2.1 Spermidine and AD

AD, also referred to as senile dementia, is characterized by progressive cognitive dysfunction and behavioral impairments. Clinically, it manifests as memory impairment, aphasia, agnosia, personality and behavioral alterations, among others. Age-associated memory decline can be attenuated by the autophagic effect of spermidine [110, 111]. Besides, spermidine has been reported to ameliorate age-related dementia [44]. It relieves mitochondrial dysfunction to maintain neuronal energy, prevent nerve cell apoptosis and inflammation as well as improve the expression of neurotrophic factors. Wirth *et al.* revealed that spermidine exerts a positive influence on memory performance among the elderly, which might be regulated by stimulating the neuromodulators in the memory system [112].

#### 3.2.2 Spermidine and PD

PD, namely paralysis agitans, is a common neurodegenerative disease that is manifested by tremors, myotonia and decreased movement abilities. Degeneration and death of dopaminergic neurons in the substantia nigra is the main pathological basis of PD. McCarty *et al.* proved that spermidine protects against PD by maintaining dopaminergic neurons functions in the mitochondria [113]. Jadiya *et al.* reported that spermidine protected against PD by inducing the *Atg* 7 dependent autophagy pathway in *C. elegans* [114]. Besides, it also protected cells in a PD model of *C. elegans* against the toxic effects through the PINK1-PDR1-dependent mitophagy pathway [115]. α-synuclein is considered to be the primary toxic trigger of PD. Previous studies found that higher spermidine concentration alleviates the process of PD through inhibition of α-synuclein and promotion of climbing activity [116, 117]. Guerra *et al.* suggested that spermidine exhibits neuroprotective effects against PD, which are mediated through its anti-inflammatory and antioxidant properties [118]. DENSPM, a polyamine analogue, and Berenil, a pharmacological agent, increases or decreases SAT1 activities, respectively. It has been confirmed that DENSPM attenuates PD histopathology while Berenil aggravates it [119].

### 3.3 Spermidine and age-related metabolic diseases

Metabolic diseases are caused by disorders in substance anabolism and catabolism, which are closely correlated with aging. Spermidine is involved in the development of metabolic diseases, such as T2DM, obesity, and metabolic syndrome [120].

#### 3.3.1 Spermidine and T2DM

T2DM is characterized by hyperglycemia due to insulin resistance. Its risk factors are complex, including aging, obesity, a strong family history of diabetes, and physical inactivity. The mechanisms and roles of spermidine in T2DM have been reported. Exogenous spermidine supplementation improves insulin sensitivity and maintains glucose homeostasis [120]. Levasseur *et al*. clarified that spermidine binds deoxyhypusine synthase (DHPS) in β cells to mRNA translation, which promotes facultative cell proliferation and glucose homeostasis maintenance [121]. Méndez *et al.* revealed that L-arginine and spermidine plays a inhibitory role in lipid peroxidation and hemoglobin glycation, which may prevent diabetic complications [122]. Besides, Wang *et al*. proved that spermidine enhanced glucose utilization through AMPK activation in myotubes, possessing a potential hypoglycemic activity in vitro [123]. Serum spermidine oxidase activity has been shown to regulate T2DM and its microvascular complications in patients [124]. Furthermore, Marx *et al.* elaborated that spermidine and agmatine were involved in renal collagen reduction in diabetic mice, thereby reducing the complications associated with diabetic nephropathy [125].

#### 3.3.2 Spermidine and obesity

Obesity is associated with multiple alterations at hormonal, inflammatory and endothelial levels, thereby enhancing morbidity rates from CVDs. In addition to its role in T2DM, spermidine was found to reduce adiposity and hepatic fat accumulation in diet-induced obese mice [120]. Besides, spermidine dietary can cause a significant weight loss and has the potential for treating obesity due to its beneficial effects in regulating in lipid metabolism, inflammatory responses, and thermogenesis [70]. Spermidine intake was negatively correlated with obesity caused by high-calorie diets and was accompanied by the induction of autophagy in white adipose tissues [126]. Notably, Ma *et al.* demonstrated that spermidine supplementation alleviated obesity in both mice and humans because its effects in enhancement of intestinal barrier functions and alteration of microbiota composition as well as functions [127]. Moreover, spermidine suppresses adiposity by inhibiting lipogenic genes expression through an AMPK-mediated mechanism [69]. Up-regulation of spermidine is accompanied by down-regulation of nicotinamide N-methyltransferase (Nnmt), which results in nicotinamide salvage regeneration of NAD^+^, increased energy expenditure, and resistance against obesity [128].

#### 3.3.3 Spermidine and metabolic syndrome

Metabolic syndrome is characterized by insulin resistance, abdominal obesity, hypertension, and hyperlipidemia. Studies have suggested an association between spermidine and metabolic syndrome. Triethylenetetramine dihydrochloride (TETA), a copper-chelator agent, is a safe pharmaceutical that can reduce obesity associated with excessive sucrose intake, high-fat diet, or leptin deficiency, since it can reduce hepatic steatosis and glucose intolerance. It has been shown that the TETA effects depended on the SAT1 activation, which can correct metabolic syndrome [129]. Moreover, Ma *et al*. confirmed that spermidine inhibited metabolic syndrome in obese mice by ameliorating hepatic steatosis and adipose tissue inflammation [70].

### 3.4 Spermidine and age-related musculoskeletal diseases

Musculoskeletal diseases are a range of degenerative and inflammatory disorders, which are a vital cause of disability. Spermidine exhibits protective roles against various musculoskeletal diseases, such as osteoporosis, sarcopenia, and osteoarthritis.

#### 3.4.1 Spermidine and osteoporosis

Osteoporosis, the most common metabolic bone disease, is characterized by microarchitectural deterioration and low bone mass. Spermidine concentration is inversely proportional to osteoporosis [130]. Spermidine dietary supplementation enhances bone strength. Besides, increased spermidine biosynthesis *in vivo* promoted warmth regeneration, which prevented bone loss through gut microbiota. Spermidine was shown to prevent against bone loss by preferentially disturbing osteoclastic activation in ovariectomized mice [131, 132]. Yeon *et al*. documented that spermidine exerts anti-osteoclastogensis and anti-migration effects by inhibiting RANKLE-mediated signaling pathway and by preventing the expression of transcription factors such as NF-κB [133].

#### 3.4.2 Spermidine and sarcopenia

Skeletal muscles are essential in inhibiting the development of multiple chronic diseases, including CVDs, T2DM, and cancer. Sarcopenia refers to age-associated progressive loss of skeletal muscle mass and function. Spermidine concentrations are associated with sarcopenia [134]. Cervelli *et al*. hypothesized that spermidine protects against aging-related skeletal muscle atrophy by regulating skeletal muscle differentiation [54]. Chrisam *et al.* revealed that systemic administration of spermidine induced autophagy in mice, leading to a concurrent amelioration of both ultrastructural and histological muscle defects [135].

#### 3.4.3 Spermidine and osteoarthritis

Osteoarthritis is one of the most prevalent and debilitating chronic joint diseases, which has been associated with a decline and loss in life quality. Sacitharan *et al*. hypothesized that spermidine is a potential therapy for osteoarthritis because it activates autophagy in osteoarthritic cartilage and reverses the reduction in polyamine synthesis [136]. Chen *et al.* showed that spermidine alleviates synovitis, osteophyte formation, and cartilage degeneration by inhibiting TNF-α induced NF-κB/p65 signaling pathway in osteoarthritis [137]. In addition, spermidine was shown to exhibit antioxidant, anti-inflammatory, and chondroprotective roles in osteoarthritic chondrocytes [138].

### 3.5 Spermidine and age-related immune diseases

Immune diseases are caused by imbalances in regulation, which affects immune responses, thereby leading to pathological changes and functional impairments. Spermidine can boost immunity. Autophagy is now recognized as an indispensable cog in the formation of long-lasting immunity, which can help host defense against viruses, bacteria, and parasites. Spermidine treatment ameliorates the decline in autophagy and reverses the senescence of old immune cell functions [31]. Puleston *et al.* found decreased T cell autophagy in elderly mice, which associated autophagy with immune-senescence [139]. They also reported that spermidine improves memory CD8+ T cell responses to influenza and cytomegalovirus infection by inducing autophagy [140]. In T cell-transfer-induced colitis models, spermidine attenuated tissue pathology by promoting homeostasis differentiation of regulatory T cells within the gut [138]. Liu *et al*. proved that dietary spermidine supplementation attenuated inflammatory bowel disease (IBD) in mice by inducing autophagy and anti-inflammatory actions associated with mitochondrial ROS-dependent AMPK activation [141].

## 4. Diagnostic and therapeutic potential of spermidine for age-related diseases

A large proportion of age-related diseases inevitably develop into functional failure or even death, because there are no drug therapies that can reverser the aging process. Therefore, early diagnosis and prompt management are particularly important. In terms of clinical applications, spermidine is a promising prognostic biomarker and a potential therapeutic agent for delaying age-related diseases.

Various pathologies are associated with elevated spermidine concentrations, promoting the possibility of spermidine being a potential disease biomarker. Soda *et al.* found a positive correlation between spermidine concentrations and CVDs-associated mortality, implying that it could be a prognostic biomarker for CVDs severity [91]. Besides, spermidine is a potential biomarker for the diagnosis of neurocognitive diseases that occur with age, such as AD or PD [31, 142]. Additionally, spermidine can be used in the diagnosis of other age-related diseases, such as cancers [143], T2DM [98], immune diseases (systemic lupus erythematosus) [144], and frailty [145] among others. However, it is worth noting that the association between elevated spermidine levels with various pathologies does not necessarily support its causal involvement.

Moving away from promising biomarker functions, spermidine is also a potential therapeutic agent for treatment of age-related diseases. Madeo *et al.* postulated that spermidine is a promising pharmaceutical intervention for aging and age-related neurodegenerative, cardiovascular, and malignant diseases [146]. Spermidine plays a protective role in aging heart, and could therefore, be used to protect against CVDs [7, 15]. A growing number of studies are reporting that spermidine is a candidate drug for neurodegenerative diseases such as AD and PD, since it can protect neurons, exert anti-inflammatory and antioxidant activities, and induce autophagy [113, 147]. In addition, there is increasing evidence that spermidine is a therapeutic agent for metabolic diseases by improving insulin sensitivity, maintaining glucose homeostasis, inhibiting lipid metabolism, and promoting thermogenesis [57, 70]. Moreover, due to its anti-inflammatory and antioxidant properties, it might be a potential therapeutic intervention for age-associated acquired immune diseases [43]. Nevertheless, the exact mechanisms have not been established and should, therefore, be evaluated further.

## 5. Conclusions and perspectives

Aging and age-related diseases share a number of basic mechanistic pillars. Aging is a catalyst in the development of age-related diseases, whereas age-related diseases exacerbate the aging process. Thus, age-induced alterations should be characterized, and aging mechanisms evaluated in order to achieve novel strategies for life extension. Spermidine regulates a wide range of biochemical and physiological aging processes and prolongs a healthy lifespan. Besides, it is a potential prognostic biomarker and therapeutic agent for evaluating and managing age-related diseases. Despite our knowledge on how spermidine brings about its anti-aging effects, there is a need for more studies. Dietary spermidine has a beneficial role in organisms. However, there exists many challenges regarding its administration to humans, including modulatory hurdles, safety and bioavailability, and clinical design issues. Remarkably, elucidation of the precise mechanisms and roles of spermidine in human aging will result in unprecedented health benefits. Therefore, studies should aim at establishing the potential causal relationship between altered spermidine metabolism and associated pathways for disease development and progression. It should also be determined whether exogenous supplementation of spermidine can delay aging, settle age-related diseases, improve life quality and eventually prolong a healthy lifespan.
